# Oral Submucous Fibrosis: A Review on Biomarkers, Pathogenic Mechanisms, and Treatments

**DOI:** 10.3390/ijms21197231

**Published:** 2020-09-30

**Authors:** Yen-Wen Shen, Yin-Hwa Shih, Lih-Jyh Fuh, Tzong-Ming Shieh

**Affiliations:** 1 School of Dentistry, China Medical University, Taichung 40402, Taiwan; a2312830@ms28.hinet.net; 2 Department of Dentistry, China Medical University Hospital, Taichung City 404332, Taiwan; 3 Department of Healthcare Administration, Asia University, Taichung 41354, Taiwan; evashih@asia.edu.tw; 4 Department of Dental Hygiene, China Medical University, Taichung 40402, Taiwan

**Keywords:** biomarkers, epidemiology, oral submucous fibrosis (OSF), pre-malignant disorders, therapeutic interventions

## Abstract

Oral submucous fibrosis (OSF) is a collagen deposition disorder that affects a patient’s oral function and quality of life. It may also potentially transform into malignancy. This review summarizes the risk factors, pathogenic mechanisms, and treatments of OSF based on clinical and bio-molecular evidence. Betel nut chewing is a major risk factor that causes OSF in Asia. However, no direct evidence of arecoline-induced carcinogenesis has been found in animal models. Despite identification of numerous biomarkers of OSF lesions and conducting trials with different drug combinations, clinicians still adopt conservative treatments that primarily focus on relieving the symptoms of OSF. Treatments focus on reducing inflammation and improving mouth opening to improve a patient’s quality of life. In conclusion, high-quality clinical studies are needed to aid clinicians in developing and applying molecular biomarkers as well as standard treatment guidelines.

## 1. Epidemiology of OSF

Oral submucous fibrosis (OSF) is a common oral precancerous lesion in Asian countries, especially in areas with a culture of chewing betel nuts. OSF is caused by abnormal collagen deposition in the connective tissues and affect mouth functions. Although there is no immediate danger with a diagnosis of OSF, it seriously affects the quality of life of patients. OSF interferes with a patient’s quality of life because of annoying symptoms, such as ulceration, xerostomia, a burning sensation, and limitation in mouth opening. Moreover, OSF is a pre-malignant disorder with the potential for malignant transformation. Therefore, it is necessary to understand its clinical features, prevalence, malignant transformation rate, and risk factors. In Asia, the major risk factor for OSF is betel nut chewing. To reduce the occurrence of OSF, it is important to discover the pre-malignant disorder in the early stage, and understand the pathologic mechanism and treatment options. This review introduces OSF from both clinical and molecular perspectives and focuses on the epidemiology, diagnostic biomarkers, mechanisms of OSF induction and transformation, and aggressive therapeutic interventions.

### 1.1. Clinical Features of OSF

The oral mucosa can be divided into masticatory, specialized, and lining mucosa based on their function and histology. OSF occurs on all three types of mucosae, and most frequently occurs in the buccal mucosa [1,2], retromolar area, and the soft palate sites. The symptoms of OSF include dry mouth, pain, taste disorders, restricted tongue mobility, trismus, dysphagia, and changed tone movability. In addition to the oral cavity, the fibrosis even involves the pharynx and esophagus. In OSF cases, the soft and pink oral mucosa initially becomes inelastic and slightly blanched. Subsequently, the mucosa becomes markedly inelastic and opaque, with white blanching, and appears papery white and tough on palpation, with a firm vertical band, which can be felt just opposite the premolar region. In the later stages, the lips and palate are also involved with lesions occurring on one or more sites. Patients with OSF experience a severe burning sensation in their mouths after ingesting spicy food. Finally, the patients’ abilities to open their mouths become limited and their oral mucosae become hardened; moreover, they have poor wound healing, and their cheeks and lips become tightly held against their teeth.

### 1.2. Prevalence of OSF

OSF is a chronic oral disease that produces scar and tissue fibrosis. It is a pre-malignant disorder that may eventually lead to oral cancer. This disease contributes significantly to mortality because of its high malignant transformation rate (1.5–15%) [3]. OSF occurrence differs with ethnicity and region and is closely associated with diet, habits, and culture [4,5,6]. South and South-East Asia have the highest prevalence of OSF patients [5,7,8]. Additionally, South Africa also has a high prevalence of OSF patients because of a large proportion of Indian immigrants. The prevalence of OSF varies among South-East Asian countries. The prevalence is reported to be 0.9–4.7% in China [9], 0.62–6.42% in India, 0.15–14.6% in Vietnam [10], and 0.086–17.6% in Taiwan [11]. Based on the World Health Organization statistics, there are more than 5 million OSF patients globally [12,13]. The ages of OSF patients range from 8 to 80 years [14], with varying mean age across different studies.

### 1.3. The Malignant Transformation Rate of OSF

OSF is widely recognized as condition precancerous to oral cancer. The malignant transformation rate of OSF ranges from 1.2 to 23% worldwide [15,16,17]. In China, epidemiologic and clinical studies have reported that the overall malignant transformation rate of OSF is 1.2 to 2.2% [9]. In India, around 7.6% of OSF patients develop oral cancer [8,18]. Based on our survey of articles available in PubMed, Google Scholar, and Medline, we found no information on the malignant transformation rate of OSF in Vietnam. The malignant transformation rate of OSF in Taiwan is about 3.27–23% [3,17,19,20]. The varying malignant transformation rate of OSF may be due to the different ages, sex, tracking period, risk factors, and pathological diagnosis of the studies. However, these studies show that OSF patients are at risk of developing oral cancers. Previous studies have proven that the duration of OSF and the degree of worsening of symptoms directly correlate with the progression to oral cancer. According to statistics, OSF generally progresses to oral cancer 3–16 years after the OSF diagnosis [15,21].

### 1.4. Risk Factors of OSF

Immunologic causes (inflammation and autoimmunity) contribute to OSF, along with nutritional factors (vitamin B, C, and iron deficiency), carcinogenic causes (chewing tobacco and betel nut), alcohol, consumption of spicy food, epigenetic regulation, and genetic predisposition [22,23,24,25,26,27]. Overconsumption of chili-containing food irritates the oral mucosa that may cause an inflammatory response to induce OSF [28]. However, in Mexico and America where chili is widely used, OSF is not found [29]. Regarding nutritional deficiency, OSF patients show significantly lower levels of serum β-carotene [30], iron [31], vitamin C [25] and, zinc [32] in a grade dependent manner; all these factors are known to negatively affect the wound healing process. Conversely, the patients also have higher serum levels of copper [32] that enhance the lysyl oxidase (LOX) activity of cross-linking collagen fibers and elastin [33]. The increase in salivary copper concentration is reported to be associated with increasing clinical grade [34]. Epigenetic alteration had been observed in Wnt inhibitory factor-1 (WIF1) [35] and p16 [36] genes of the buccal cells in OSF patients. Hypermethylation of these two genes also contributes to the potential malignancy of OSF.

Current epidemiological studies and evidence indicate that betel nut chewing is one of the most significant risk factors for OSF [37,38]. Unfortunately, the commercially modified areca nut is cheap, sweet, and readily available in India. Moreover, the low oral health literacy of parents and children furthers the risk of addiction in young children, which consequently increases the burden of OSF [39,40]. Arecoline is the major compound in betel nut that initiates the OSF process [41,42,43]. Low doses of arecoline enhance the cell proliferation rate, while high doses of arecoline induce total reactive oxygen species (ROS), DNA damage, and LOX activity [44]. The ataxia-telangiectasia mutated activated DNA repair is also inhibited by arecoline [45]. LOX is overexpressed in oral cancer and upregulated by arecoline treatment of cells [46,47,48].

Among OSF patients in China, 62.3–99.85% have a habit of chewing betel nuts [9,49,50]. Men had a significantly higher OSF prevalence than women. Betel quid is composed of halved betel nut with dried flavoring substances. Bags of different brands of betel quid are sold in the markets of China [9]. In India, areca nut-based chewing substrates were used by patients in 219 of 220 OSF cases (99.55%) [5] and the incidence of OSF was more common in women than in men, possibly because of the significant differences in chewing habits between men and women (with men chewing gutkha, mawa, and kharra, and women chewing only areca nut) [51]. Another study demonstrated that the male-to-female ratio in OSF was 4.9:1. OSF occurred in a significantly younger age group among men than that in women [51]. Betel quid chewing is still prevalent in Vietnam, but there is no detailed information on its association with OSF. The average male-to-female ratio among OSF patients was 1.05. The prevalence of oral pre-cancerous lesions and other oral mucosal diseases was significantly higher in the betel quid chewing group (80.4%) [10]. In Taiwan, where betel quid does not have the tobacco additive, 85.4–100% OSF patients have betel nut chewing habits [52,53], and 73.4% patients with betel nut chewing-induced OSF swallow the juice of betel quid during the chewing process [53]. The average male-to-female ratio among OSF patients was 8.52. Men had a significantly higher OSF prevalence than women because most of the betel nut users in Taiwan are men [11].

In addition to chewing betel nut, some studies reported that habits such as chewing tobacco, smoking, and drinking alcohol increase the probability of developing OSF [24,54]. An observational study of 1000 cases in India showed that a high percentage of men chewed areca nut combined with tobacco [51]. A study in Taiwan indicated that a high proportion of betel quid chewers are also smokers (86%) or drinkers (74%) [9]. The combination of chewing betel nut and tobacco has led to an obvious increase in the frequency of OSF [55]. Other studies have also confirmed that consuming and chewing betel nut have an additive effect on the development of OSF [9,52].

## 2. Differential Diagnosis of OSF

Understanding the progression of the OSF can help to determine the appropriate treatment. Because of advancements in biotechnology, the research on OSF biomarkers has become more and more vigorous, which is helpful for the diagnosis of OSF and malignant transformation.

### 2.1. OSF Classification by Function

Various staging/grading classification systems of OSF have been documented in the past, such as clinical, functional, and histological staging/classification of OSF. Most systems include three staging/grading classifications according to the mucosa status, fibrous bands, and mouth opening [55,56,57]. Some of the staging systems are used by doctors in the clinic to diagnose or treat OSF [22,58]. According to clinical staging/classification, patients with early OSF show stomatitis and vesiculation; those with moderate OSF show a marble-like appearance and palpable fibrous bands; and those with severe OSF show leukoplakia and erythroplakia in the lesion. The maximum average mouth opening of 18–30 years is 56.60 mm for men and 51.04 mm for women in healthy individuals [59]. On functional staging/classification of OSF, the maximum interincisal mouth openings are divided into >20 mm, 11–19 mm, and <10 mm for stage 1 to stage 3, respectively (Table 1) [55].

### 2.2. OSF Classification by Histology

It is worth noting that the OSF can transform to oral squamous cell carcinoma (OSCC), and that solid biopsy is needed for clinical diagnosis and treatment. In histological staging/classification, the amount and distribution of fibroblasts, collagen fibers, inflammatory cells, and blood vessels is used to determine the various stages of OSF [60]. Generally, OSF is classified into four histopathological stages (Table 2) [61,62]. The pathological characteristics are chronic inflammation and excessive collagen deposition in the connective tissues below the oral mucosal epithelium, accompanying local inflammation in the lamina propria or deep connective tissues, and degenerative changes in muscles. Generally, epithelial atrophy [62,63], and loss of rete pegs [64] are also reported. The sub-epithelium of patients with OSF show fibrosis with chronic inflammatory cells, such as lymphocytes, monocytes, plasma cells, and macrophages. Dense collagen bundles caused hyalinized areas in the connective tissue, and the vascularity and lumen were reduced in the connective tissue.

### 2.3. Biomarkers of OSF

Some underlying molecular differences exist between normal, oral, and potentially malignant disorders and early and late cancer stages. In recent years, biomarkers have been developed through existing biology techniques; cytological features, promoter methylation, polymorphism, mRNAs, microRNA, non-coding RNAs, and protein and trace elements in a solid biopsy, liquid biopsy from serum, and saliva have been used as potential biomarkers for OSF (Table 3) [65]. The use of cytology, tissue, serum, and saliva samples for analysis has unique advantages. It is easy to collect and observe mucosal cells with appropriate staining methods. Although histology is the most accurate method for examination, it is invasive, with long waiting times for test reports and low patient acceptance. Recently, the development of body fluid biopsy testing showed less invasiveness, shorter waiting times for testing reports, and high patient acceptance, although more supporting data are needed to establish accuracy. There are more biomarkers in typical solid biopsy studies than in liquid biopsy currently. These biomarkers have not been widely used in OSF stage classification. Larger sample sizes are needed to establish the accuracy of most novel biomarkers. At present, clinical symptoms and pathological examination are still the mainstay of OSF diagnosis. Combining instrumentation with biomarkers is a more effective method to save time and reagents in OSF diagnosis. In addition to the diagnosis of OSF, single or multiple biomarkers expression levels may also be used as a new OSF staging method for improvement of OSF evaluation index, evaluate OSF transformation to malignant tumor index, and for gene and targeted therapy.

## 3. The Mechanism of OSF Pathogenesis and Malignant Transformation

OSF is prevalent in Asia, and the main risk factor is betel nut chewing. The clinical biomarkers indicated the inflammation reaction and collagen deposition disorder contribute to OSF. Clinical data showed identical biomarkers in late-stage OSF and malignant transformation tissues. It may interpret some parts of the mechanisms of how OSF transform into malignancy. In this section, we integrated the clinical findings and animal studies to have a profound understanding of the etiology.

### 3.1. Pathogenesis of OSF

OSF is mainly induced by areca nut chewing in Asia. The main components of areca nut contain 31.1% phenols, 18.7% polysaccharides, 14% fat, 10.8% fiber, and 0.5% alkaloids [103]. Arecoline is the main alkaloid that causes the pathogenesis of the OSF [38,39,40]. Arecoline stimulates the fibroblast cells to express growth factors and cytokines that enhance the collagen deposition and repress the collagen degradation. Clinical studies reported transforming growth factor beta (TGF-β), connective tissue growth factor (CTGF), beta fibroblast growth factor (bFGF) [104], alpha-smooth muscle actin (α-SMA) [105], tumor necrosis factor-α (TNF-α) [106], serum c-reactive protein [107], ROS level [108], matrix metalloproteinases (MMP), and the tissue inhibitors of metalloproteinases (TIMP) [109] were expressed abnormally in OSF group.

Arecoline activates the oral tissue express TNF-α that stimulates cell inflammation. Cell inflammation will activate the wound healing reaction, which decreases MMP and increases TIMP expression. This TIMP and MMP expression profile is also found in the oral tissue of OSF patients [109]. The function of MMP is to degrade the extracellular matrix protein, and TIMP inhibits this process. This contributes to the abnormal collagen deposition on the lesion. Inflammation reaction also stimulates the cell express bFGF and TGFβ-1. Continuously overexpressing bFGF in oral cells contributes to the collagen deposition disorder in OSF [104]. TGFβ-1 stimulates fibroblasts to transform to myofibroblasts, which are mainly responsible for collagen production [110,111] and wound contraction. Normally, myofibroblasts undergo apoptosis after finishing the mission of wound healing [112]. However, this mechanism is disrupted in OSF. Arecoline also increases the ROS level in OSF patients’ serum [108]. Serum ROS attacks the structure of the blood vessels in endothelial cells, induced cell senescence [113], and DNA double-stranded breaks [114]. The decrease in blood flow around the oral mucosa finally causes one of the pathological symptoms - epithelial atrophy. The cell inflammation reaction and ROS attack stimulate the cell to activate the TGF-β signaling pathways. The TGF-β signaling is responsible for ceasing the cell cycle and promoting apoptosis in the unrepaired damage cells while the cells are damaged by stimulants. TGF-β also activates the downstream gene, CTGF, expression [115] to promote the fibroblast-mediated production of extracellular matrix deposition [116].

In addition, copper participates in the cross-linking of collagen [117]. It enhances the hardness of the oral submucosa tissue and exacerbates the limitation of mouth opening and trismus [118]. The commercial areca nut was reported to contain a significantly higher level of copper than the raw areca nut [117]. Higher serum copper was also found among OSF patients [119] and was deemed as one of the factors that induce OSF.

### 3.2. Transformation of OSF to Malignancy

OSF is deemed as a pre-malignant phenotype of OSCC. However, a cross-sectional multi-central study in Pakistan, from 2004 to 2012, showed that among 1774 patients, 26.6% had malignant transformation of OSF to OSCC, and 30.27% had OSCC without clinically visible OSF [120]. Another study reported the potential malignant rate of OSF to be 7–30% [121]. This reveals that one-third of OSF cases have a chance to turn into malignancy. The mechanism underlying the transformation of OSF to OSCC is still unclear. Researchers conducted a clinical observational study to identify molecular biomarkers from patient specimens and detect a possible link between OSF and OSCC. The common molecular markers in late-stage OSF and OSCC were cysteine proteinase inhibitor [122], TGF-β1 [123], hypoxia-inducible factor 1α (HIF-1α) [124], DNA damage phenotype [125], MMP and TIMP [126], and Cytokeratin [127] at the lesion site and elevated serum immunoglobulin G (IgG) [128]. Common alterations of the epigenetic regulation are also reported in late-stage OSF and OSCC. The hypermethylation of WIF1, p16, which was mentioned in Section 1.4, and the expression of long-noncoding RNA HCG22, RP11-397A16.1, LINC00271, CTD-3179P9.1, and ZNF667-AS1 [129] have been reported and may contribute to OSF malignant development.

Wound healing is a process that helps the human body repair tissue damage. However, persistent inflammation, collagen deposition, growth factors, and cytokine secretion induced by arecoline may lead to malignancy. TGF-β activation in late-stage cancer can promote tumorigenesis, including metastasis and chemoresistance [130]. CTGF is involved in epithelial-mesenchymal transition and angiogenesis [116]. TNF activates distinct signaling pathways to decide the cell fate [131]; the Nuclear factor-κB (NF-κB) pathway contributes to cell survival, and the c-Jun N-terminal kinase pathway contributes to cell death. TNF-α is reported to stimulate cancer cell growth, proliferation, invasion, and metastasis [83]. MMPs have been reported as one of the main factors of cancer progression and metastasis formation [132].

Carcinogenesis has four main phases, including initiation, promotion, progression, and metastasis. Normal cells are exposed to carcinogens that induced DNA damage, as well as dysregulate cellular proliferation, survival, differentiation, and the DNA repair function [133]. Areca nut, tobacco, and alcohol are the three main carcinogens in oral cancer. In vitro and in vivo studies have revealed that arecoline and areca alkaloid induced mutagenicity in vitro and carcinogenicity in vivo [134]. A clinical observational study showed the potential for malignant transformation of OSF cases in individuals consuming both areca nut and tobacco consumption [51]. The promotion stage is a lengthy and irreversible process. This is a phase between a pre-malignant lesion and the development of malignant tumors. Once the patient suffers from OSF, limitation of mouth opening and burning sensation compel them to seek medical help. Medical intervention and eliminating the use of the carcinogens effectively interrupts the malignant promotion process. This may explain why the clinical malignant transformation rate of OSF is at most 30% rather than 100%. Clinical studies also proved that prolong areca nut and tobacco use induced malignant transformation [51].

From the other point of view, change of the microenvironment around the fibrosis tissue is also a malignant promotion factor. While the collagen deposition alters in oral mucosa, the compact tissue oppresses the capillaries and block the blood flow that produces a hypoxic environment suitable for the promotion of malignant cell growth [135]. Virus infection may be one factor that induces OSF malignancy [136]. Human papillomavirus types 16 and 18 are well-known viruses that cause oral cancer. OSF patients have a higher infection rate of human papillomavirus (HPV) than the normal group, which could explain the potential malignancy of OSF [137].

### 3.3. OSF and Malignancy Formation-The Evidence on Animal Models

Building an animal model is crucial for investigating the mechanism of OSF formation and malignant transformation. Early in 1997, Huan et al. established an animal model using Sprague-Dawley (SD) rats; they either injected or applied aqueous areca nut extracts (AANE) to buccal mucosa and successfully induced the collagen deposition in the buccal mucosa [138]. The common animal species used in OSF animal models are SD rats and BALB/C mice. Usually, males are used to avoid the effects of hormone fluctuations. All the studies use chemicals in areca nut as a material, including areca nut water extract and arecoline (Table 4). These studies are all conducted in Asia. Some studies inject the water extract into the subcutaneous region, while others topically applied the water extract onto the surface of buccal mucosa followed by fasting for 2 hrs. Other studies added the water extract into drinking bottles. The dosage depends on the purity of the compound and the method of administration. Topical arecoline applied at 8 mg/mL could effectively induce OSF. If the AANE is administered through a water bottle, the concentration is much higher.

The animal models successfully mimic the phenotype of human OSF, including limitation of mouth opening [139] and the elevation of biomarkers TGF-β1 [140] and type I and type III collagen [139]. However, we have still not observed malignant transformation in arecoline-induced OSF animal models, possibly because of insufficient dosage or treatment duration or owing to the biological differences between humans and mice/rats. Numerous studies have effectively induced malignancy formation using arecoline combined with 4-Nitroquinoline-1-oxide or benzo a pyrene [141,142]. This type of animal model mimics a clinical patient who consumes both the areca nut and tobacco.

Moreover, oral intake of areca nut powder had been confirmed a rapid systematic absorption of areca nut alkaloids in human [134]. We have known that some metabolic disease like diabetes is associated with betel nut chewing [143]. The arecoline-induced OSF animal model does not merely provide the synergism test of arecoline with other carcinogens like 4-Nitroquinoline-1-oxide and benzo a pyrene but also an in-depth exploration of arecoline-induced systematic disease for future studies. At present, there is no direct evidence to indicate arecoline-induced carcinogenesis in rat/mice oral mucosa, which makes clinical drug discovery for target therapy of arecoline-induced potential malignancy challenging. The scientists need to develop better in vitro cell models and in vivo animal models to study the key factors of OSF malignant transformation, and more samples and evidence are needed to support them. These studies will be the translational medical basis for future OSF drug development, gene therapy and targeted therapy.

## 4. Treatment Strategy

OSF contributes to the hardness of submucosa tissue. It jointly affects the muscles, bones, and joints movement below the submucosa tissue, which will eventually affect the degree of mouth opening and results in trismus. Limitation of the mouth opening makes it difficult in daily routine oral cleaning, speaking, and eating. Combining the annoying symptoms like burning sensation and xerostomia, patients’ quality of life is low. The goals of clinical treatments are relieving the annoying symptoms and improving mouth opening to elevate the patients’ quality of life. The current treatment methods for OSF are mainly divided into three categories: drug treatment, mouth opening exercising, and elective surgery. However, there is no standardized treatment protocol for clinicians. In this section, we summarize the commonly used drugs, types of exercise devices, and types of surgeries mentioned in the recent literature.

### 4.1. Clinical Drug Treatments of OSF

As mentioned in Section 3, the main clinical phenotypes of OSF are inflammation and fibrosis on the oral mucosa. Clinical drug treatment mainly focuses on resolving the inflammation and fibrosis in the oral tissue. Some adjuvants, such as vitamins, minerals, and vasodilators, help ameliorate the sign and symptoms.

Corticosteroids [147], such as hydrocortisone, triamcinolone, dexamethasone, and betamethasone, as well as anti-inflammatory cytokines, such as interferon-gamma (IFN-γ) [148], ameliorate inflammation and decrease collagen formation. Enzymatic drugs, such as collagenase, hyaluronidase, and chymotrypsin [2], have been used in OSF treatment. These enzymes reduce the viscosity of the extracellular matrix and show good improvement in patients with trismus and fibrosis [149,150]. Supplementation of vitamins and minerals are reported to effectively ameliorate the burning sensation and ulceration [151]. Table 5 summarizes the commonly used drugs.

We were able to find numerous articles investigating the drug treatment effect of OSF in online databases. Most of them aim to relieve the symptoms of mouth opening limitation and burning sensation. Studies are attempting different combinations of corticosteroids with an adjuvant to investigate the OSF treatment effect. However, the combination treatment may present risks. Many clinical studies examined the conventional treatment of combining steroid injections and hyaluronidase with topical vitamin A, steroid applications, and oral iron preparations found. These studies found a hazardous effect where only the conservative treatment of the steroid injections steroids in combination with hyaluronidase was found to be safe [152]. To our knowledge, no articles provided a standard guideline and advised the most appropriate drug combination for the management of OSF. We agree with the point of view of Prof. Hu (2010) [153]. High-quality clinical studies are needed to help clinicians develop a standard treatment guideline for OSF drug treatment.

### 4.2. Mouth Exercising Devices

Therapeutic conventional exercise [155,156] is a commonly used and non-invasive treatment method for patients with OSF. The devices can be prefabricated like EZBite or custom-made like an oral stent. At present, most devices are designed for vertical oral movement. The devices developed by P. G. Pati and S. P. Patil in 2012 squeeze/stretch the cheek to increase elasticity and blood circulation of the oral mucosa [157]. Mouth exercising devices can significantly improve the mouth opening by approximately 10.5 mm and maintain these results for 12 weeks to 6 months [158]. Other commercially available devices such as TheraBite^®^, Malmö, Sweden Jaw Motion, Rehabilitation System™ (TheraBite), and Dynasplint Trismus System^®^ (DTS), have been reported to increase mouth opening up to 14 mm [159]. EZBite designed by Li et al. in 2019, provides users a clear and simple protocol and continuously trains patients to open their mouths to a certain quantitative value each time to achieve maximal interincisal opening from 15.7 to 29.7 mm [156,160].

Although various types of devices are available for this treatment option, there is still no consensus standard for the frequency or duration of use, or how much strength should be given to the fibrotic tissue. However, these mouth exercising devices are not suitable for individuals with periodontal disease and with a partially edentulous ridge on the anterior arches, which causes soft tissue injuries [158] and pain [161].

### 4.3. Elective Surgery

Surgery is required for patients with severe OSF whose mouth opening is less than 20 mm. Clinicians use scalpel blades, electrocautery, and lasers to cut the fibers that restrict mouth opening, and coronoidectomy to reconstruct soft tissue to increase mouth opening. The fat flap, nasolabial flap, tongue flap, mandibular mucoperiosteal flap, palatal flap, and platysma myocutaneous flap [162] are used for soft tissue reconstruction. The flap must be derived from well-vascularized tissue close to the defect, with minimum donor site morbidity to prevent OSF recurrence. A total of 150 patients in 1995 underwent buccal fat pad grafting; it was particularly successful in diminishing scarring after two years as compared with split-thickness skin and fresh human amnion grafts [163]. Medical treatment with a vasodilator with antioxidant, anti-inflammatory, and immune-modulatory properties was used after flap transplantation [164].

### 4.4. Comparison of Treatment Methods

Treatment of OSF with clinical drugs primarily includes injecting the treatment direction to the local lesion to infiltrate the drugs deep into the tissue. The efficacy of oral medication may interfere with the patient’s other prescription drugs. The efficacy of oral ointment may interfere with the constitution of saliva. For mild symptoms, drugs are the recommended first-line prescription, and the patient’s history of medication must be taken into consideration [165].

Mouth exercising is the most acceptable treatment method for patients owing to its low cost, convenience, and non-invasiveness. However, treatment efficacy depends on the patient’s self-motivation. Self-motivation can increase by using biofeedback app, which tracks changes in patients and increases their motivation [166]. While patients choose using mouth exercising devices, the intensive exercise protocol and fitting problems between the patient and the devices must be evaluated first [159]. It can be constructed by 3D printing technology, oral scanning equipment, and CT images to provide a customized instrument.

For OSF patients undergoing complex surgery in severe late stages, scalpel blades, electrocautery, and laser therapy are alternative choices for the treatment of OSF. Laser therapy provides a choice that will not disappoint clinicians or patients. It limits the collagen damage up to 5 μm that makes a bloodless therapy, minimizing tissue shrinkage, without damage teeth, nerves and no wound suturing [162]. Treatment by laser, Diode, KTP 532, and ErCr: YSGG, for OSF management produce good results with no limitations on age, gender, ethnicity, or socioeconomic status of the participants. The most important thing is that the use of lasers can increase cheek flexibility. However, continuous mouth opening exercises are needed to maintain the treatment effects postoperatively. Sixty-two percent of individuals in a study underwent surgical excision with fresh amnion graft, which decreased inter-ID in the range of 5–10 mm after a two-year follow-up [163]. We summarized the comparison outcomes of treatment methods in Table 6.

## 5. Conclusions

The prevalence of OSF and the rate of malignant transformation are different among countries. Quitting betel nut chewing is the best strategy to prevent OSF and potential malignancy. Regardless of the strategy, clinical diagnosis and treatment are still based on conservative methods. The treatment must improve the elasticity of the oral mucosa and mouth opening distance. This ensures that patients have normal oral functions like speaking and eating to improves the patient’s quality of life and provides an adequate nutritional intake. High-quality clinical studies are needed to help clinicians to develop and apply molecular biomarkers and to formulate standard treatment guidelines for OSF.

## Figures and Tables

**Table 1:** Oral submucous fibrosis (OSF) staging/classification system.

Clinical Stage	Functional Stage
Stage I: faucial bands only	Stage A: mouth opening >20 mm
Stage II: faucial and buccal bands	Stage B: mouth opening 11–19 mm
Stage III: faucial, buccal and labial bands	Stage C: mouth opening <10 mm

**Table 2:** Histological staging of oral submucous fibrosis [62].

Group	Histological Features
Group I-very early	Fine fibrillar collagen network interspersed with marked edema, blood vessels dilated and congested, large aggregate of plump fibroblasts with abundant cytoplasm, inflammatory cells mainly PMN with few eosinophils. Epithelium normal, with occasional hyperplasia
Group II-early	Juxta-epithelial hyalinization with collagen present as thickened but separate bundles, blood vessels dilated and congested, moderate number of young fibroblasts, inflammatory cells mainly PMN, eosinophils, and occasional plasma cells. Epithelium shows flattening/shortening of rete pegs with varying degree of keratinization
Group III-moderately advanced	Juxta-epithelial hyalinization is present. Faintly discernible collagen bundles separated by very slight, residual edema. Muscle fibers interspersed within collagen fibers reveal the beginning of degeneration and irregularity of striae. Blood vessels constricted, mature fibrocytes with scanty cytoplasm, and spindle-shaped nuclei. Inflammatory cells, mainly lymphocytes and plasma cells. Epithelium markedly atrophic with total loss of rete pegs
Group IV-advanced	Collagen hyalinized as a smooth sheet eliminating all evidence of individual bundles. Extensive fibrosis obliterating the mucosal blood vessels and eliminating melanocytes. Fibroblasts markedly absent within hyalinized zones. Extensive degeneration of muscle fibers. Total loss of rete pegs with mild-to-moderate atypia

PMN: Polymorphonuclear.

**Table 3:** Biomarkers in OSF specimens.

Specimens		Upregulation	Downregulation
Cells	Cytology	micronuclei in exfoliated buccal cells [66]	
Tissues	DNA	hyper-methylated loci reported in three or more studies included p16, p14, MGMT and DAPK [67]	
		Wnt inhibitory factor-1 promoter methylation [35]	
		secreted frizzled-related proteins (SFRP-1) and SFRP-5 [68]	
		Matrix metalloproteinases-3 (MMP3) polymorphism [69]	
	RNA	transforming growth factor beta receptor (TGF-βR1) and TGF-βR2 [70]	miR-200b [71]
		LncRNA LINC00974 [72,73]	miR-200c [74]
		miR-199-5p [75]	miR-203 [76,77]
		miR-1246 [78]; miR10-b [79]	LncRNA GAS5-AS1 [80]
	Protein	TGF-βR1 and TGF-βR2 [70]	β-catenin [81]
		proliferating cell nuclear antigen (PCNA) [82]	Wnt inhibitory factor-1 (WIF1) [35]
		cyclophilin A [83,84]	SFRP-1 and SFRP-5 [68]
		nuclear coactivator 7 (NCOA7) [85]	
		hypoxia-inducible factor 1α (*HIF*-1α) and plasminogen activator inhibitor-1 (PAI-1) [86]	
		endoglin (CD105) [87]	
		Ki67, cyclin D1, mesenchymal-epithelial transition factor (c-Met), insulin-like growth factor II mRNA binding protein 3 (IMP3) [81]	
		β-catenin [68]	
		α-enolase [88]	
		Zinc finger E-box-binding homeobox 1 (ZEB1) [74]	
		ZEB2 [71]	
Serum	Cytology	sister chromatid exchange in lymphocytes [89]	
		DNA damage in lymphocyte [90]	
	Protein	lactate dehydrogenase (LDH) [91,92]	superoxide dismutase (SOD) and glutathione peroxidase (GPx) [93]
		malondialdehyde (MDA) [90]	serum protein, globulin [94]
	Others	Copper [95]	β-carotene [96]
Saliva	RNA	S100A7 [97,98,99]	
	Protein	LDH [91,92,100]	GPx and SOD [101]
	Others	8-hydroxy-2-deoxyguanosine (8-OHdG) and MDA [102]	vitamin C and vitamin E [102]

**Table 4:** Summary of the arecoline-induced OSF animal models.

Author/Year/Country	Animal Type	Methods	Outcomes	Ref.
		Drug, Dosage, Control, Observed Time Point	Phenotypes or Markers	
M.W. Sumeth Perera/2007/Sri Lanka	Female BALB/c 10–12 weeks of age Weighing 28–30 g	Topical application of aqueous areca nut extracts 256 mg/mL on buccal mucosa twice daily 6 days per week Control group: apply 50 mM NaCl. Observed at 300th, 350th, 450th and 600th day	Epithelial atrophy, Increased cellularity of fibroblasts, Fibrosis of connective tissue, Focal infiltration of inflammatory cells, Muscle atrophy.	[144]
M.H. Chiang/2016/Taiwan	Specific pathogen-free male BALB/C mice 6-week weeks of age Weighing 20 g	Subcutaneous (SC) injection of 100μL ANE, 10mg/mL and 20 mg/mL on mice shaving back once per 2 day. Control group, PBS injection Observed on 3rd, 7th, 14th,30th day	Increased dermal thickness Collagen deposition, Elevated biomarkers: α-SMA, and connective tissue growth factor (CTGF)	[145]
Shilpa Maria/2016/India	Sprague-Dawley rats Weighing 120–150 g	Inject o.2ml supernatant of o.2 g/6 mL water areca nut extract to buccal mucosa every alternate day for 48 weeks Control group: inject 0.2 mL saline Observed duration: every 6 weeks	OSF-like lesions: Atrophic epithelium, Inflammation and accumulation of dense bundles of collagen fibers Upregulation of TGF-β1	[140]
Qi-Tao Wen/2017/China	Male BALB/c mouse (6 weeks old)	Mice were allowed to drink distilled water containing1000 mg/mL arecoline Control group: drinking distilled water Replace water bottle once a week Observed every 2 weeks to 20 weeks	Epithelium atrophy, Elevated collagen type I and angiogenesis	[146]
Bo Yang/2019/China	Male SD rat Weighing 325.4±22.7 g	Arecoline 0.5, 2, 8 mg/mL dissolved in normal saline and wiping on oral mucosa with cotton bud plus mechanical stimulation Control group, w/o mechanical stimulation Once per 2 days Observed at 16th week.	Mouth openings were significantly reduced, and the expression levels of type Ⅲ collagen and TGF-β1 were significantly increased. Mechanical stimulation can increase the three indexes of mucosa, no pathological change and difference in the mouth opening was observed.	[139]

**Table 5:** The common drugs in clinical drug treatment of OSF.

Drug Name	Drug Type	Effect	Reference
Hydrocortisone, Triamcinolone, Dexamethasone Betamethasone	Corticosteroid	Anti-inflammation	[147]
IFN-γ	Cytokines	Anti-inflammation	[148]
Collagenase, Hyaluronidase	Enzymes	Breaking down the ground substance in connective tissue	[149] [150]
Pentoxifylline	Vasodilator	Hampered mucosal vascularity	[154]
Isoxsuprine			
Vitamin A, B, C, D, E	Adjuvant	Deactivate free radicals	[151]
Iron,	Adjuvant	Relief the symptom	[25]

**Table 6:** Comparison of different treatment methods for OSF.

Items	Drug Treatments	Mouth Exercising Devices	Elective Surgery
Long recovery time for wound	+	+	+++
Increase mouth opening	+	++	+++
Side effect	++	+	+++
Cost	++	+	+++
Need for patient cooperation	+	+++	++

Score sort: +: Lowest ++: Medium +++: Highest.

## References

[1] Dionne K.R., Warnakulasuriya S., Zain R.B., Cheong S.C. (2015). Potentially malignant disorders of the oral cavity: Current practice and future directions in the clinic and laboratory. Int. J. Cancer.

[2] Chole R.H., Gondivkar S.M., Gadbail A.R., Balsaraf S., Chaudhary S., Dhore S.V., Ghonmode S., Balwani S., Mankar M., Tiwari M. (2012). Review of drug treatment of oral submucous fibrosis. Oral Oncol..

[3] Wang Y.Y., Tail Y.H., Wang W.C., Chen C.Y., Kao Y.H., Chen Y.K., Chen C.H. (2014). Malignant transformation in 5071 southern Taiwanese patients with potentially malignant oral mucosal disorders. BMC Oral Health.

[4] Zhang X., Reichart P.A. (2007). A review of betel quid chewing, oral cancer and precancer in Mainland China. Oral Oncol..

[5] Tilakaratne W.M., Ekanayaka R.P., Warnakulasuriya S. (2016). Oral submucous fibrosis: A historical perspective and a review on etiology and pathogenesis. Oral Surg. Oral Med. Oral Pathol. Oral Radiol..

[6] Chattopadhyay A., Ray J.G. (2016). Molecular Pathology of Malignant Transformation of Oral Submucous Fibrosis. J. Environ. Pathol. Toxicol. Oncol..

[7] Chang M.C., Chiang C.P., Lin C.L., Lee J.J., Hahn L.J., Jeng J.H. (2005). Cell-mediated immunity and head and neck cancer: With special emphasis on betel quid chewing habit. Oral Oncol..

[8] Cox S.C., Walker D.M. (1996). Oral submucous fibrosis. A review. Aust. Dent. J..

[9] Liu B., Shen M., Xiong J., Yuan Y., Wu X., Gao X., Xu J., Guo F., Jian X. (2015). Synergistic effects of betel quid chewing, tobacco use (in the form of cigarette smoking), and alcohol consumption on the risk of malignant transformation of oral submucous fibrosis (OSF): A case-control study in Hunan Province, China. Oral Surg. Oral Med. Oral Pathol. Oral Radiol..

[10] Reichart P.A., Nguyen X.H. (2008). Betel quid chewing, oral cancer and other oral mucosal diseases in Vietnam: A review. J. Oral Pathol. Med. Off. Publ. Int. Assoc. Oral Pathol. Am. Acad. Oral Pathol..

[11] Yang S.F., Wang Y.H., Su N.Y., Yu H.C., Wei C.Y., Yu C.H., Chang Y.C. (2018). Changes in prevalence of precancerous oral submucous fibrosis from 1996 to 2013 in Taiwan: A nationwide population-based retrospective study. J. Med. Assoc..

[12] Nigam N.K., Aravinda K., Dhillon M., Gupta S., Reddy S., Srinivas Raju M. (2014). Prevalence of oral submucous fibrosis among habitual gutkha and areca nut chewers in Moradabad district. J. Oral Biol. Craniofac. Res..

[13] Gottipamula S., Sundarrajan S., Moorthy A., Padmanabhan S., Sridhar K.N. (2018). Buccal Mucosal Epithelial Cells Downregulate CTGF Expression in Buccal Submucosal Fibrosis Fibroblasts. J. Maxillofac. Oral Surg..

[14] Maher R., Lee A.J., Warnakulasuriya K.A., Lewis J.A., Johnson N.W. (1994). Role of areca nut in the causation of oral submucous fibrosis: A case-control study in Pakistan. J. Oral Pathol. Med. Off. Publ. Int. Assoc. Oral Pathol. Am. Acad. Oral Pathol..

[15] Pindborg J.J., Murti P.R., Bhonsle R.B., Gupta P.C., Daftary D.K., Mehta F.S. (1984). Oral submucous fibrosis as a precancerous condition. Scand. J. Dent. Res..

[16] Murti P.R., Bhonsle R.B., Pindborg J.J., Daftary D.K., Gupta P.C., Mehta F.S. (1985). Malignant transformation rate in oral submucous fibrosis over a 17-year period. Community Dent. Oral Epidemiol..

[17] Shiau Y.Y., Kwan H.W. (1979). Submucous fibrosis in Taiwan. Oral Surg. Oral Med. Oral Pathol..

[18] Tang J.G., Jian X.F., Gao M.L., Ling T.Y., Zhang K.H. (1997). Epidemiological survey of oral submucous fibrosis in Xiangtan City, Hunan Province, China. Community Dent. Oral Epidemiol..

[19] Lian Ie B., Tseng Y.T., Su C.C., Tsai K.Y. (2013). Progression of precancerous lesions to oral cancer: Results based on the Taiwan National Health Insurance Database. Oral Oncol..

[20] Yang P.Y., Chen Y.T., Wang Y.H., Su N.Y., Yu H.C., Chang Y.C. (2017). Malignant transformation of oral submucous fibrosis in Taiwan: A nationwide population-based retrospective cohort study. J. Oral Pathol. Med. Off. Publ. Int. Assoc. Oral Pathol. Am. Acad. Oral Pathol..

[21] Jeng J.H., Chang M.C., Hahn L.J. (2001). Role of areca nut in betel quid-associated chemical carcinogenesis: Current awareness and future perspectives. Oral Oncol..

[22] Passi D., Bhanot P., Kacker D., Chahal D., Atri M., Panwar Y. (2017). Oral submucous fibrosis: Newer proposed classification with critical updates in pathogenesis and management strategies. Natl. J. Maxillofac. Surg..

[23] Balakrishnan C., Aswath N. (2015). Estimation of serum, salivary immunoglobulin G, immunoglobulin A levels and total protein, hemoglobin in smokeless tobacco chewers and oral submucous fibrosis patients. Contemp. Clin. Dent..

[24] Arakeri G., Rai K.K., Hunasgi S., Merkx M.A.W., Gao S., Brennan P.A. (2017). Oral submucous fibrosis: An update on current theories of pathogenesis. J. Oral Pathol. Med. Off. Publ. Int. Assoc. Oral Pathol. Am. Acad. Oral Pathol..

[25] Guruprasad R., Nair P.P., Singh M., Singh M., Singh M., Jain A. (2014). Serum vitamin c and iron levels in oral submucous fibrosis. Indian J. Dent..

[26] Wang Y.P., Wu Y.C., Cheng S.J., Chen H.M., Sun A., Chang J.Y. (2015). High frequencies of vitamin B12 and folic acid deficiencies and gastric parietal cell antibody positivity in oral submucous fibrosis patients. J. Formos. Med. Assoc. = Taiwan Yi Zhi.

[27] Teh M.T., Tilakaratne W.M., Chaplin T., Young B.D., Ariyawardana A., Pitiyage G., Lalli A., Stewart J.E., Hagi-Pavli E., Cruchley A. (2008). Fingerprinting genomic instability in oral submucous fibrosis. J. Oral Pathol. Med. Off. Publ. Int. Assoc. Oral Pathol. Am. Acad. Oral Pathol..

[28] Seedat H.A., van Wyk C.W. (1988). Submucous fibrosis in non-betel nut chewing subjects. J. Biol. Buccale.

[29] Ratheesh A.V., Kumar B., Mehta H., Sujatha G.P., Shankarmurthy S.P. (2015). Etiopathogenesis of oral submucous fibrosis. J. Med. Radiol. Pathol. Surg..

[30] Raina C., Raizada R.M., Chaturvedi V.N., Harinath B.C., Puttewar M.P., Kennedy A.K. (2005). Clinical profile and serum beta-carotene levels in oral submucous fibrosis. Indian J. Otolaryngol. Head Neck Surg..

[31] Thakur M., Guttikonda V.R. (2017). Estimation of hemoglobin, serum iron, total iron-binding capacity and serum ferritin levels in oral submucous fibrosis: A clinicopathological study. J. Oral Maxillofac. Pathol..

[32] Sachdev P.K., Freeland-Graves J., Beretvas S.N., Sanjeevi N. (2018). Zinc, Copper, and Iron in Oral Submucous Fibrosis: A Meta-Analysis. Int. J. Dent..

[33] Vallet S.D., Ricard-Blum S. (2019). Lysyl oxidases: From enzyme activity to extracellular matrix cross-links. Essays Biochem..

[34] Mohammed F., Manohar V., Jose M., Thapasum A.F., Mohamed S., Shamaz B.H., D’Souza N. (2015). Estimation of copper in saliva and areca nut products and its correlation with histological grades of oral submucous fibrosis. J. Oral Pathol. Med. Off. Publ. Int. Assoc. Oral Pathol. Am. Acad. Oral Pathol..

[35] Zhou S., Chen L., Mashrah M., Zhu Y., He Z., Hu Y., Xiang T., Yao Z., Guo F., Zhang C. (2015). Expression and promoter methylation of Wnt inhibitory factor-1 in the development of oral submucous fibrosis. Oncol. Rep..

[36] Kaliyaperumal S., Sankarapandian S. (2016). Evaluation of p16 hypermethylation in oral submucous fibrosis: A quantitative and comparative analysis in buccal cells and saliva using real-time methylation-specific polymerase chain reaction. South Asian J. Cancer.

[37] Tilakaratne W.M., Klinikowski M.F., Saku T., Peters T.J., Warnakulasuriya S. (2006). Oral submucous fibrosis: Review on aetiology and pathogenesis. Oral Oncol..

[38] Angadi P.V., Rekha K.P. (2011). Oral submucous fibrosis: A clinicopathologic review of 205 cases in Indians. Oral Maxillofac. Surg..

[39] Jain A., Taneja S. (2019). Oral Submucous Fibrosis in Pediatric Patients: A Systematic Review and Protocol for Management. Int. J. Surg. Oncol..

[40] Deshpande A., Kiran S., Dhillon S., Mallikarjuna R. (2013). Oral submucous fibrosis: A premalignant condition in a 14-year-old Indian girl. BMJ Case Rep..

[41] Li J., Yao M., Zhu X., Li Q., He J., Chen L., Wang W., Zhu C., Shen T., Cao R. (2019). YAP-Induced Endothelial-Mesenchymal Transition in Oral Submucous Fibrosis. J. Dent. Res..

[42] Hsieh Y.P., Wu K.J., Chen H.M., Deng Y.T. (2018). Arecoline activates latent transforming growth factor beta1 via mitochondrial reactive oxygen species in buccal fibroblasts: Suppression by epigallocatechin-3-gallate. J. Formos. Med. Assoc. = Taiwan Yi Zhi.

[43] Tsai C.H., Yang S.F., Chen Y.J., Chu S.C., Hsieh Y.S., Chang Y.C. (2004). Regulation of interleukin-6 expression by arecoline in human buccal mucosal fibroblasts is related to intracellular glutathione levels. Oral Dis..

[44] Tu H.F., Chen M.Y., Lai J.C., Chen Y.L., Wong Y.W., Yang C.C., Chen H.Y., Hsia S.M., Shih Y.H., Shieh T.M. (2019). Arecoline-regulated ataxia telangiectasia mutated expression level in oral cancer progression. Head Neck.

[45] Shih Y.H., Chiu K.C., Wang T.H., Lan W.C., Tsai B.H., Wu L.J., Hsia S.M., Shieh T.M. (2020). Effects of melatonin to arecoline-induced reactive oxygen species production and DNA damage in oral squamous cell carcinoma. J. Formos. Med. Assoc. = Taiwan Yi Zhi.

[46] Wang T.H., Hsia S.M., Shieh T.M. (2016). Lysyl Oxidase and the Tumor Microenvironment. Int. J. Mol. Sci..

[47] Shih Y.H., Chang K.W., Chen M.Y., Yu C.C., Lin D.J., Hsia S.M., Huang H.L., Shieh T.M. (2013). Lysyl oxidase and enhancement of cell proliferation and angiogenesis in oral squamous cell carcinoma. Head Neck.

[48] Shieh T.M., Lin S.C., Liu C.J., Chang S.S., Ku T.H., Chang K.W. (2007). Association of expression aberrances and genetic polymorphisms of lysyl oxidase with areca-associated oral tumorigenesis. Clin. Cancer Res..

[49] Zhang S.S., Li W.H., Gao Y.J., Liu Z.W., Liu L., Tang J.Q., Ling T.Y. (2012). Betel-quid and oral submucous fibrosis: A cross-sectional study in Hunan province, China. J. Oral Pathol. Med. Off. Publ. Int. Assoc. Oral Pathol. Am. Acad. Oral Pathol..

[50] Cai X., Yao Z., Liu G., Cui L., Li H., Huang J. (2019). Oral submucous fibrosis: A clinicopathological study of 674 cases in China. J. Oral Pathol. Med. Off. Publ. Int. Assoc. Oral Pathol. Am. Acad. Oral Pathol..

[51] Hazarey V.K., Erlewad D.M., Mundhe K.A., Ughade S.N. (2007). Oral submucous fibrosis: Study of 1000 cases from central India. J. Oral Pathol. Med..

[52] Lee C.H., Ko Y.C., Huang H.L., Chao Y.Y., Tsai C.C., Shieh T.Y., Lin L.M. (2003). The precancer risk of betel quid chewing, tobacco use and alcohol consumption in oral leukoplakia and oral submucous fibrosis in southern Taiwan. Br. J. Cancer.

[53] Chiang C.P., Hsieh R.P., Chen T.H., Chang Y.F., Liu B.Y., Wang J.T., Sun A., Kuo M.Y. (2002). High incidence of autoantibodies in Taiwanese patients with oral submucous fibrosis. J. Oral Pathol. Med..

[54] Aishwarya K.M., Reddy M.P., Kulkarni S., Doshi D., Reddy B.S., Satyanarayana D. (2017). Effect of Frequency and Duration of Tobacco Use on Oral Mucosal Lesions—A Cross-Sectional Study among Tobacco Users in Hyderabad, India. Asian Pac. J. Cancer Prev..

[55] Haider S.M., Merchant A.T., Fikree F.F., Rahbar M.H. (2000). Clinical and functional staging of oral submucous fibrosis. Br. J. Oral Maxillofac. Surg..

[56] Rajendran R., George T. (2003). Morphohistometric analysis of advancing tumor fronts in malignancies associated with oral submucous fibrosis. Indian J. Dent. Res. Off. Publ. Indian Soc. Dent. Res..

[57] Pindborg J.J. (1989). Oral submucous fibrosis: A review. Ann. Acad. Med. Singap..

[58] Gondivkar D.S.M., Gadbail D.A.R., Sarode D.S.C., Gondivkar D.R.S., Patil S., Gaikwad D.R.N., Dinh-Toi C., Yuwanati D.M. (2020). Treatment outcomes of laser therapy in oral submucous fibrosis-a systematic review. J. Oral Biol. Craniofac. Res..

[59] Mezitis M., Rallis G., Zachariades N. (1989). The Normal Range of Mouth Opening. J. Oral Maxil. Surg..

[60] Reshma V., Varsha B.K., Rakesh P., Radhika M.B., Soumya M., D’Mello S. (2016). Aggrandizing oral submucous fibrosis grading using an adjunct special stain: A pilot study. J. Oral Maxillofac. Pathol..

[61] Khanna J.N., Andrade N.N. (1995). Oral submucous fibrosis: A new concept in surgical management. Report of 100 cases. Int. J. Oral Maxillofac. Surg..

[62] Bhatt P., Manjunath M., Khakhla D., Gubrellay P., Bhargava R., Guruprasad L. (2019). Assessment and correlation between functional and histological staging of oral submucous fibrosis: A clinicohistopathologic study. Natl. J. Maxillofac. Surg..

[63] Ranganathan K., Devi M.U., Joshua E., Kirankumar K., Saraswathi T.R. (2004). Oral submucous fibrosis: A case-control study in Chennai, South India. J. Oral Pathol. Med. Off. Publ. Int. Assoc. Oral Pathol. Am. Acad. Oral Pathol..

[64] Holla V.A., Chatra L.K., Shenai P., Shetty D., Baliga A. (2016). A Study to Analyze Different Patterns of Quid Usage among Subjects with Oral Submucous Fibrosis in Mangalore Population. Adv. Med..

[65] Shih Y.H., Wang T.H., Shieh T.M., Tseng Y.H. (2019). Oral Submucous Fibrosis: A Review on Etiopathogenesis, Diagnosis, and Therapy. Int. J. Mol. Sci..

[66] Gupta N., Rakshit A., Srivastava S., Suryawanshi H., Kumar P., Naik R. (2019). Comparative evaluation of micronuclei in exfoliated oral epithelial cells in potentially malignant disorders and malignant lesions using special stains. J. Oral Maxillofac. Pathol..

[67] Shridhar K., Walia G.K., Aggarwal A., Gulati S., Geetha A.V., Prabhakaran D., Dhillon P.K., Rajaraman P. (2016). DNA methylation markers for oral pre-cancer progression: A critical review. Oral Oncol..

[68] Zhou S., Chen L., Mashrah M., Zhu Y., Liu J., Yang X., He Z., Wang L., Xiang T., Yao Z. (2015). Deregulation of secreted frizzled-related proteins is associated with aberrant beta-catenin activation in the carcinogenesis of oral submucous fibrosis. Onco Targets.

[69] Zade P.R., Gosavi S.R., Hazarey V.K., Ganvir S.M. (2017). Matrix metalloproteinases-3 gene-promoter polymorphism as a risk factor in oral submucous fibrosis in an Indian population: A pilot study. J. Investig. Clin. Dent..

[70] Rai A., Ahmad T., Parveen S., Parveen S., Faizan M.I., Ali S. (2020). Expression of transforming growth factor beta in oral submucous fibrosis. J. Oral Biol. Craniofac. Res..

[71] Liao Y.W., Yu C.C., Hsieh P.L., Chang Y.C. (2018). miR-200b ameliorates myofibroblast transdifferentiation in precancerous oral submucous fibrosis through targeting ZEB2. J. Cell Mol. Med..

[72] Lin C.Y., Hsieh P.L., Liao Y.W., Peng C.Y., Yu C.C., Lu M.Y. (2019). Arctigenin Reduces Myofibroblast Activities in Oral Submucous Fibrosis by LINC00974 Inhibition. Int. J. Mol. Sci..

[73] Fang C.Y., Yu C.C., Liao Y.W., Hsieh P.L., Lu M.Y., Lin K.C., Wu C.Z., Tsai L.L. (2019). LncRNA LINC00974 activates TGF-beta/Smad signaling to promote oral fibrogenesis. J. Oral Pathol. Med. Off. Publ. Int. Assoc. Oral Pathol. Am. Acad. Oral Pathol..

[74] Lu M.Y., Yu C.C., Chen P.Y., Hsieh P.L., Peng C.Y., Liao Y.W., Yu C.H., Lin K.H. (2018). miR-200c inhibits the arecoline-associated myofibroblastic transdifferentiation in buccal mucosal fibroblasts. J. Med. Assoc..

[75] Yuan Y., Li N., Zeng L., Shen Z., Jiang C. (2019). Pathogenesis investigation of miR-199-5p in oral submucous fibrosis based on bioinformatics analysis. Oral Dis..

[76] Chen J., Liu B.J., Du C., Cao Q., Li M., Feng H. (2016). [Target regulation of micro-RNA-203 to the expression of collagen type alpha 4 and its role in oral submucous fibrosis]. Zhonghua Kou Qiang Yi Xue Za Zhi = Zhonghua Kouqiang Yixue Zazhi = Chin. J. Stomatol..

[77] Zheng L., Jian X., Guo F., Li N., Jiang C., Yin P., Min A.J., Huang L. (2015). miR-203 inhibits arecoline-induced epithelial-mesenchymal transition by regulating secreted frizzled-related protein 4 and transmembrane-4 L six family member 1 in oral submucous fibrosis. Oncol. Rep..

[78] Liu C.M., Liao Y.W., Hsieh P.L., Yu C.H., Chueh P.J., Lin T., Yang P.Y., Yu C.C., Chou M.Y. (2019). miR-1246 as a therapeutic target in oral submucosa fibrosis pathogenesis. J. Formos. Med. Assoc. = Taiwan Yi Zhi.

[79] Fang C.Y., Yu C.C., Liao Y.W., Hsieh P.L., Ohiro Y., Chu P.M., Huang Y.C., Yu C.H., Tsai L.L. (2020). miR-10b regulated by Twist maintains myofibroblasts activities in oral submucous fibrosis. J. Formos. Med. Assoc. = Taiwan Yi Zhi.

[80] Lin C.Y., Liao Y.W., Hsieh P.L., Lu M.Y., Peng C.Y., Chu P.M., Yang H.W., Huang Y.F., Yu C.C., Yu C.H. (2018). LncRNA GAS5-AS1 inhibits myofibroblasts activities in oral submucous fibrosis. J. Formos. Med. Assoc. = Taiwan Yi Zhi.

[81] Bazarsad S., Zhang X., Kim K.Y., Illeperuma R., Jayasinghe R.D., Tilakaratne W.M., Kim J. (2017). Identification of a combined biomarker for malignant transformation in oral submucous fibrosis. J. Oral Pathol. Med..

[82] Keshav R., Narayanappa U. (2015). Expression of Proliferating Cell Nuclear Antigen (PCNA) in Oral Submucous Fibrosis: An Immunohistochemical Study. J. Clin. Diagn. Res..

[83] Tang D., Tao D., Fang Y., Deng C., Xu Q., Zhou J. (2017). TNF-Alpha Promotes Invasion and Metastasis via NF-Kappa B Pathway in Oral Squamous Cell Carcinoma. Med. Sci. Monit. Basic Res..

[84] Yuan Y., Hou X., Feng H., Liu R., Xu H., Gong W., Deng J., Sun C., Gao Y., Peng J. (2016). Proteomic identification of cyclophilin A as a potential biomarker and therapeutic target in oral submucous fibrosis. Oncotarget.

[85] Xie X., Jiang Y., Yuan Y., Wang P., Li X., Chen F., Sun C., Zhao H., Zeng X., Jiang L. (2016). MALDI imaging reveals NCOA7 as a potential biomarker in oral squamous cell carcinoma arising from oral submucous fibrosis. Oncotarget.

[86] Tsai C.H., Lee S.S., Chang Y.C. (2015). Hypoxic regulation of plasminogen activator inhibitor-1 expression in human buccal mucosa fibroblasts stimulated with arecoline. J. Oral Pathol. Med. Off. Publ. Int. Assoc. Oral Pathol. Am. Acad. Oral Pathol..

[87] Pammar C., Nayak R.S., Kotrashetti V.S., Hosmani J. (2018). Comparison of microvessel density using CD34 and CD105 in oral submucous fibrosis and its correlation with clinicopathological features: An immunohistochemical study. J. Cancer Res..

[88] Bag S., Dutta D., Chaudhary A., Sing B.C., Pal M., Ray A.K., Banerjee R., Paul R.R., Basak A., Das A.K. (2018). Identification of alpha-enolase as a prognostic and diagnostic precancer biomarker in oral submucous fibrosis. J. Clin. Pathol..

[89] Jeyapradha D., Saraswathi T., Ranganathan K., Wilson K. (2011). Comparison of the frequency of sister chromatid exchange in pan chewers and oral submucous fibrosis patients. J. Oral Maxillofac. Pathol..

[90] Paulose S., Rangdhol V., Ramesh R., Jeelani S.A., Brooklyin S. (2016). Estimation of serum malondialdehyde and assessment of DNA damage using comet assay in patients with oral submucous fibrosis. J. Investig. Clin. Dent..

[91] Sivaramakrishnan M., Sivapathasundharam B., Jananni M. (2015). Evaluation of lactate dehydrogenase enzyme activity in saliva and serum of oral submucous fibrosis patients. J. Oral Pathol. Med..

[92] Mishra S., Kritika C., Bajoria A.A., Choudhury P., Sahoo S.K., Sangamesh N.C. (2018). Estimation of Salivary and Serum Lactate Dehydrogenase in Oral Submucous Fibrosis. J. Int. Soc. Prev. Community Dent..

[93] Gurudath S., Ganapathy K.S., Pai A., Ballal S., Asha M.L. (2012). Estimation of superoxide dismutase and glutathione peroxidase in oral submucous fibrosis, oral leukoplakia and oral cancer--a comparative study. Asian Pac. J. Cancer Prev..

[94] More C.B., Shah P.H., Venkatesh R. (2017). Estimation of Serum Protein in Oral Potentially Malignant Disorders and Oral Malignancy—A Cross-Sectional Study. J. Clin. Diagn. Res..

[95] Hosthor S.S., Mahesh P., Priya S.A., Sharada P., Jyotsna M., Chitra S. (2014). Quantitative analysis of serum levels of trace elements in patients with oral submucous fibrosis and oral squamous cell carcinoma: A randomized cross-sectional study. J. Oral Maxillofac. Pathol..

[96] Rathod Y.G., Kulkarni S.P., Khairnar M.R., Joshi P.N., Patle B.K., Pagare J.S. (2018). Estimation of serum beta-carotene level in patients suffering from oral submucous fibrosis. J. Exp. Oncol..

[97] Raffat M.A., Hadi N.I., Hosein M., Zubairi A.M., Ikram S., Akram Z. (2019). Differential expression of salivary S100A7 in oral submucous fibrosis. Saudi. Dent. J..

[98] Zhou G., Xie T.X., Zhao M., Jasser S.A., Younes M.N., Sano D., Lin J., Kupferman M.E., Santillan A.A., Patel V. (2008). Reciprocal negative regulation between S100A7/psoriasin and beta-catenin signaling plays an important role in tumor progression of squamous cell carcinoma of oral cavity. Oncogene.

[99] Kaur J., Matta A., Kak I., Srivastava G., Assi J., Leong I., Witterick I., Colgan T.J., Macmillan C., Siu K.W. (2014). S100A7 overexpression is a predictive marker for high risk of malignant transformation in oral dysplasia. Int. J. Cancer.

[100] Kallalli B.N., Rawson K., Muzammil, Singh A., Awati M.A., Shivhare P. (2016). Lactate dehydrogenase as a biomarker in oral cancer and oral submucous fibrosis. J. Oral Pathol. Med. Off. Publ. Int. Assoc. Oral Pathol. Am. Acad. Oral Pathol..

[101] Divyambika C.V., Sathasivasubramanian S., Vani G., Vanishree A.J., Malathi N. (2018). Correlation of Clinical and Histopathological Grades in Oral Submucous Fibrosis Patients with Oxidative Stress Markers in Saliva. Indian J. Clin. Biochem..

[102] Kaur J., Politis C., Jacobs R. (2016). Salivary 8-hydroxy-2-deoxyguanosine, malondialdehyde, vitamin C, and vitamin E in oral pre-cancer and cancer: Diagnostic value and free radical mechanism of action. Clin. Oral Investig..

[103] Zhang Y., Zhang J.J., Kang W.Y., Yan W.Y. (2014). [Advances of chemical constituents and pharmacological activities of Myristica genus]. Zhongguo Zhong Yao Za Zhi.

[104] Bishen K.A., Radhakrishnan R., Satyamoorthy K. (2008). The role of basic fibroblast growth factor in oral submucous fibrosis pathogenesis. J. Oral Pathol. Med..

[105] Gabbiani G., Chaponnier C., Huttner I. (1978). Cytoplasmic filaments and gap junctions in epithelial cells and myofibroblasts during wound healing. J. Cell Biol..

[106] Shinde A.V., Humeres C., Frangogiannis N.G. (2017). The role of alpha-smooth muscle actin in fibroblast-mediated matrix contraction and remodeling. Biochim. Biophys. Acta Mol. Basis Dis..

[107] Gosavi S.R., Torkadi A.A. (2020). Serum C-reactive protein in oral submucous fibrosis and oral squamous cell carcinoma: A cross-sectional study. J. Oral Maxillofac. Pathol..

[108] Shakunthala G.K., Annigeri R.G., Arunkumar S. (2015). Role of oxidative stress in the pathogenesis of oral submucous fibrosis: A preliminary prospective study. Contemp. Clin. Dent..

[109] Rajendran R., Rajeesh M.P., Shaikh S., Shanthi, Pillai M.R. (2006). Expression of matrix metalloproteinases (MMP-1, MMP-2 and MMP-9) and their inhibitors (TIMP-1 and TIMP-2) in oral submucous fibrosis. Indian J. Dent. Res..

[110] Lee I.T., Lin C.C., Wu Y.C., Yang C.M. (2010). TNF-alpha induces matrix metalloproteinase-9 expression in A549 cells: Role of TNFR1/TRAF2/PKCalpha-dependent signaling pathways. J. Cell Physiol..

[111] Carswell E.A., Old L.J., Kassel R.L., Green S., Fiore N., Williamson B. (1975). An endotoxin-induced serum factor that causes necrosis of tumors. Proc. Natl. Acad. Sci. USA.

[112] Hinz B. (2007). Formation and function of the myofibroblast during tissue repair. J. Investig. Derm..

[113] Davalli P., Mitic T., Caporali A., Lauriola A., D’Arca D. (2016). ROS, Cell Senescence, and Novel Molecular Mechanisms in Aging and Age-Related Diseases. Oxid. Med. Cell. Longev..

[114] Bautista-Nino P.K., Portilla-Fernandez E., Vaughan D.E., Danser A.H., Roks A.J. (2016). DNA Damage: A Main Determinant of Vascular Aging. Int. J. Mol. Sci..

[115] Arnott J.A., Lambi A.G., Mundy C., Hendesi H., Pixley R.A., Owen T.A., Safadi F.F., Popoff S.N. (2011). The role of connective tissue growth factor (CTGF/CCN2) in skeletogenesis. Crit. Rev. Eukaryot. Gene Exp..

[116] Lipson K.E., Wong C., Teng Y., Spong S. (2012). CTGF is a central mediator of tissue remodeling and fibrosis and its inhibition can reverse the process of fibrosis. Fibrogenesis Tissue Repair..

[117] Opsahl W., Zeronian H., Ellison M., Lewis D., Rucker R.B., Riggins R.S. (1982). Role of copper in collagen cross-linking and its influence on selected mechanical properties of chick bone and tendon. J. Nutr..

[118] Tom A., Baghirath V., Krishna B., Ganepalli A., Kumar J.V., Mohan S.P. (2019). Ultrastructural Changes of Collagen in Different Histopathological Grades of Oral Submucous Fibrosis. J. Pharm. Bioallied. Sci..

[119] Yadav A., Kumar L., Misra N., Deepak U., Shiv Kumar G.C. (2015). Estimation of serum zinc, copper, and iron in the patients of oral submucous fibrosis. Natl. J. Maxillofac. Surg..

[120] Mohiuddin S., Fatima N., Hosein S., Hosein M. (2016). High risk of malignant transformation of oral submucous fibrosis in Pakistani females: A potential national disaster. J. Pak. Med. Assoc..

[121] Bari S., Metgud R., Vyas Z., Tak A. (2017). An update on studies on etiological factors, disease progression, and malignant transformation in oral submucous fibrosis. J. Cancer Res..

[122] Anila Namboodiripad P.C. (2014). Cystatin C: Its role in pathogenesis of OSMF. J. Oral Biol. Craniofac. Res..

[123] Krishnamurthy V.K.P.S.R. (2014). Transforming growth factor beta 1 in oral submucous fibrosis: An immunohistochemical study—Understanding the pathogenesis. J. Dent. Res. Rev..

[124] Pandiar D., Shameena P. (2014). Immunohistochemical expression of CD34 and basic fibroblast growth factor (bFGF) in oral submucous fibrosis. J. Oral Maxillofac. Pathol..

[125] Udupa R., Hallikeri K., Trivedi D.J. (2014). The comet assay a method to measure DNA damage in oral submucous fibrosis patients: A case-control study. Clin. Cancer Investig. J..

[126] Shrestha A., Carnelio S. (2013). Evaluation of matrix metalloproteinases-2 (MMP-2) and tissue inhibitors of metalloproteinases-2 (TIMP-2) in oral submucous fibrosis and their correlation with disease severity. Kathmandu Univ. Med. J. (Kumj).

[127] Ranganathan K., Kavitha R., Sawant S.S., Vaidya M.M. (2006). Cytokeratin expression in oral submucous fibrosis--an immunohistochemical study. J. Oral Pathol. Med..

[128] Divya V.C., Sathasivasubramanian S. (2014). Estimation of serum and salivary immunoglobulin G and immunoglobulin A in oral pre-cancer: A study in oral submucous fibrosis and oral lichen planus. J. Nat. Sci. Biol. Med..

[129] Zhou S., Zhu Y., He Z., Zhang D., Guo F., Jian X., Zhang C. (2019). Long Non-Coding RNA Expression Profile Associated with Malignant Progression of Oral Submucous Fibrosis. J. Oncol..

[130] Colak S., Ten Dijke P. (2017). Targeting TGF-beta Signaling in Cancer. Trends Cancer.

[131] Wang X., Lin Y. (2008). Tumor necrosis factor and cancer, buddies or foes?. Acta Pharm. Sin..

[132] Mishev G., Deliverska E., Hlushchuk R., Velinov N., Aebersold D., Weinstein F., Djonov V. (2014). Prognostic value of matrix metalloproteinases in oral squamous cell carcinoma. Biotechnol. Biotechnol. Equip..

[133] Siddiqui I.A., Sanna V., Ahmad N., Sechi M., Mukhtar H. (2015). Resveratrol nanoformulation for cancer prevention and therapy. Ann. N. Y. Acad. Sci..

[134] IARC Working Group on the Evaluation of Carcinogenic Risks to Humans (2004). Betel-quid and areca-nut chewing and some areca-nut derived nitrosamines. IARC Monogr. Eval. Carcinog. Risks Hum..

[135] Murgod V.V., Kale A.D., Angadi P.V., Hallikerimath S. (2014). Morphometric analysis of the mucosal vasculature in oral submucous fibrosis and its comparison with oral squamous cell carcinoma. J. Oral Sci..

[136] Martin-Hernan F., Sanchez-Hernandez J.G., Cano J., Campo J., del Romero J. (2013). Oral cancer, HPV infection and evidence of sexual transmission. Med. Oral Patol. Oral Cir. Bucal..

[137] Jalouli J., Ibrahim S.O., Mehrotra R., Jalouli M.M., Sapkota D., Larsson P.A., Hirsch J.M. (2010). Prevalence of viral (HPV, EBV, HSV) infections in oral submucous fibrosis and oral cancer from India. Acta Otolaryngol..

[138] Huang S., Ling T., Wu H. (1997). [Experimental study on aqueous areca nut extracts inducing oral submucous fibrosis in rats. I. Observation of histomorphology]. Hua Xi Kou Qiang Yi Xue Za Zhi.

[139] Yang B., Fu M.F., Tang Z.G. (2019). [Rat model with oral submucous fibrosis induced by arecoline and mechanical stimulation]. Hua Xi Kou Qiang Yi Xue Za Zhi.

[140] Maria S., Kamath V.V., Satelur K., Rajkumar K. (2016). Evaluation of transforming growth factor beta1 gene in oral submucous fibrosis induced in Sprague-Dawley rats by injections of areca nut and pan masala (commercial areca nut product) extracts. J. Cancer Res..

[141] Chang N.W., Pei R.J., Tseng H.C., Yeh K.T., Chan H.C., Lee M.R., Lin C., Hsieh W.T., Kao M.C., Tsai M.H. (2010). Co-treating with arecoline and 4-nitroquinoline 1-oxide to establish a mouse model mimicking oral tumorigenesis. Chem. Biol. Interact..

[142] Huang J.L., Lu H.H., Lu Y.N., Hung P.S., Lin Y.J., Lin C.C., Yang C.C., Wong T.Y., Lu S.Y., Lin C.S. (2016). Enhancement of the genotoxicity of benzo[a]pyrene by arecoline through suppression of DNA repair in HEp-2 cells. Toxicol. Vitr..

[143] Tseng C.H. (2008). Betel nut chewing is associated with hypertension in Taiwanese type 2 diabetic patients. Hypertens. Res..

[144] Sumeth Perera M.W., Gunasinghe D., Perera P.A., Ranasinghe A., Amaratunga P., Warnakulasuriya S., Kaluarachchi K. (2007). Development of an in vivo mouse model to study oral submucous fibrosis. J. Oral Pathol. Med..

[145] Chiang M.H., Chen P.H., Chen Y.K., Chen C.H., Ho M.L., Wang Y.H. (2016). Characterization of a Novel Dermal Fibrosis Model Induced by Areca Nut Extract that Mimics Oral Submucous Fibrosis. PLoS ONE.

[146] Wen Q.T., Wang T., Yu D.H., Wang Z.R., Sun Y., Liang C.W. (2017). Development of a mouse model of arecoline-induced oral mucosal fibrosis. Asian Pac. J. Trop. Med..

[147] Tilakaratne W.M., Ekanayaka R.P., Herath M., Jayasinghe R.D., Sitheeque M., Amarasinghe H. (2016). Intralesional corticosteroids as a treatment for restricted mouth opening in oral submucous fibrosis. Oral Surg. Oral Med. Oral Pathol. Oral Radiol..

[148] Haque M.F., Meghji S., Nazir R., Harris M. (2001). Interferon gamma (IFN-gamma) may reverse oral submucous fibrosis. J. Oral Pathol. Med..

[149] Lin H.J., Lin J.C. (2007). Treatment of oral submucous fibrosis by collagenase: Effects on oral opening and eating function. Oral Dis..

[150] James L., Shetty A., Rishi D., Abraham M. (2015). Management of Oral Submucous Fibrosis with Injection of Hyaluronidase and Dexamethasone in Grade III Oral Submucous Fibrosis: A Retrospective Study. J. Int. Oral Health.

[151] Maher R., Aga P., Johnson N.W., Sankaranarayanan R., Warnakulasuriya S. (1997). Evaluation of multiple micronutrient supplementation in the management of oral submucous fibrosis in Karachi, Pakistan. Nutr. Cancer.

[152] Borle R.M., Borle S.R. (1991). Management of oral submucous fibrosis: A conservative approach. J. Oral Maxillofac. Surg..

[153] Angadi P.V. (2010). Drug treatment for oral submucous fibrosis. Evid. Based Dent..

[154] Rajendran R., Rani V., Shaikh S. (2006). Pentoxifylline therapy: A new adjunct in the treatment of oral submucous fibrosis. Indian J. Dent. Res..

[155] Dani V.B., Patel S.H. (2018). The effectiveness of therapeutic ultrasound in patients with oral submucosal fibrosis. Indian J. Cancer.

[156] Li Y.H., Chang W.C., Chiang T.E., Lin C.S., Chen Y.W. (2019). Mouth-opening device as a treatment modality in trismus patients with head and neck cancer and oral submucous fibrosis: A prospective study. Clin. Oral Investig..

[157] Patil P.G., Patil S.P. (2012). Novel mouth-exercising device for oral submucous fibrosis. J. Prosthodont..

[158] Gondivkar S.M., Gadbail A.R., Sarode S.C., Gondivkar R.S., Patil S., Gaikwad R.N., Yuwanati M. (2020). Clinical efficacy of mouth exercising devices in oral submucous fibrosis: A systematic review. J. Oral Biol. Craniofac. Res..

[159] van der Geer S.J., Reintsema H., Kamstra J.I., Roodenburg J.L.N., Dijkstra P.U. (2020). The use of stretching devices for treatment of trismus in head and neck cancer patients: A randomized controlled trial. Support Care Cancer.

[160] Li Y.H., Liu C.C., Chiang T.E., Chen Y.W. (2018). EZBite open-mouth device: A new treatment option for oral submucous fibrosis-related trismus. J. Dent. Sci..

[161] Cox S., Zoellner H. (2009). Physiotherapeutic treatment improves oral opening in oral submucous fibrosis. J. Oral Pathol. Med. Off. Publ. Int. Assoc. Oral Pathol. Am. Acad. Oral Pathol..

[162] Kapre M., Sudhanshu K. (2018). Surgery of Trismus in Oral Submucous Fibrosis. https://www.springer.com/gp/book/9789811048906.

[163] Lai D.R., Chen H.R., Lin L.M., Huang Y.L., Tsai C.C. (1995). Clinical-Evaluation of Different Treatment Methods for Oral Submucous Fibrosis—a 10-Year Experience with 150 Cases. J. Oral Pathol. Med..

[164] Kholakiya Y., Jose A., Rawat A., Nagori S.A., Jacob S., Roychoudhury A. (2020). Surgical management of oral submucous fibrosis with “Seagull-nasolabial flap” combined with short-term oral pentoxifylline for preventing relapse. J. Stomatol. Oral Maxillofac. Surg..

[165] Panta P., Sarode S.C., Sarode G.S., Patil S. (2018). New Directions for Oral Submucous Fibrosis Research: Whole Evaluation for Holistic Rehabilitation!. J. Contemp. Dent. Pr..

[166] Maia A.V., Furlan R., Moraes K.O., Amaral M.S., Medeiros A.M., Motta A.R. (2019). Tongue strength rehabilitation using biofeedback: A case report. Codas.

